# On the Continued Growth of the Incisors in Rodents, and Their Reproduction, Considered in Reference to Their Application to the Study of the Comparative Anatomy of the Teeth, Preceded by Some New Researches upon the Origin and Development of the Dental Follicles

**Published:** 1860-01

**Authors:** J. E. Oudet

**Affiliations:** Docteur en Médecine, Membre de l'Academie Nationale de Médecine, etc., etc.


### ARTICLE VI.

*On the Continued Growth of the Incisors in Rodents, and their Reproduction, considered in Reference to their application to the Study of the Comparative Anatomy of the Teeth, preceded by some New Researches upon the Origin and Development of the Dental Follicles.* By J. E. ODUDET, Docteur en Médecine, Membre de l'Academie Nationale de Médecine, etc., etc. Translated for the American Journal of Dental Science.

### *On the Continued Growth of the Incisors in Rodents.*

#### 1.—*Origin of the Dental Follicles. Epoch of their Appearance.*

RELYING upon the light which experimental physiology and comparative anatomy have furnished, I have demonstrated, during the past twenty-five years, that studied in respect to the organic acts which preside at their formation, the teeth belong to the mucous system like skin, horns, hair, nails, etc. Now, I shall show that the same analogy exists in the first anatomical disposition of the organs which produce these divers substances.

The follicles which accompany the two dentitions are not formed at the same time, and do not effect the same arrangement. Toward the commencement of the third month after conception, each cavity of the two jaws contains four sacs, of which two anterior and two posterior, adoss equally by pairs one against the other so as to leave a tolerably large interval between the two anterior and the two posterior sacs. The first are the smallest and appertain to the temporary incisors ; the others belong to the molars of the same class (temporary.) At the last of the third month, towards the middle and behind the interval which we have indicated and which is marked by a prominence of the external layer of the maxillary bone, a fifth sac, for the canine, may be discovered, which completes the whole number of the follicles for the temporary teeth.

The development of the follicles of second dentition is announced, towards the last of the fourth month, by the appearance of a sixth sac at the bottom of the depression or groove which represent the interior of the jaws. This sac appertains to the first large molar. The capsules of the secondary incisors are only distinctly perceivable, during the seventh month, and still later those of the canine. At the period of birth, all these little sacs exist, glued against the follicles of the temporary teeth ; it is necessary, it may be here stated, to take some precautions in order to discover them. The best way is to remove the internal layer of the maxillary bones ; we then find, at the superior part and posterior to the sacs of the incisors and of the temporary canines, an equal number of other little sacs which correspond to them and which we soon recognize, after having incised the posterior face of their capsules, to be the follicles of the incisors and permanent canines. These sacs are more elevated than the others, and are situated very near the gums with which it is easy to remove them, if attempted with care.

The pulp of the permanent incisors is then very much developed and its configuration entirely marked out. That of the canine is much smaller and less advanced. These sacs are only separated from the follicles of the temporaries by a very slight fibrous layer and are contained in the same alveolar cavities. As to the follicles of the bicuspids which M. Serres says he has proved the existence of in the fœtus, and which he indicates as situated under the germs of the molars of the first dentition, notwithstanding the numerous dissections which I have made, I have never been able to meet with except at a much more advanced period. The follicle of the anterior bicuspid, ordinarily preceded by several months by that of the second permanent large molar, can be perceived at the last of the second year, but oftener during the third, and is soon followed by the follicle of the posterior bicuspid, these follicles of the bicuspids moreover, have primitively the same relations to the gums as the follicles of the incisors and permanent canines. At first they adhere very slightly but equally to the internal border of this membrane; they acquire additional volume, however, gradually ; they elongate and seem as if suspended to the gums by a very delicate and short cord formed by their external envelop which will eventually, by a greater extension, constitute their appendage. At this epoch, they correspond to the internal face of the roots of the milk molars vis-a-vis to the interval which separates them ; as dentition proceeds they continue to descend toward the bottom of the alveoli and finally arrive at the bottom of the vault formed by the roots of the teeth. I have never found the follicle of the third large molars, at least distinctly, before the age of nine or ten years and sometimes later. However, on following the nervous filament, which is intended for these teeth, in the jaws of young children, I sometime since recognized and published* that, at the place where it joins the gum, I had remarked a swelling having some resemblance to a nervous ganglion ; but I only dared to affirm that this was the follicle of the wisdom tooth. These facts have not remained sterile, and soon I shall make some new researches to which they may conduct me.

Such are the dental follicles as they appear, in the interior of the jaw, when they have acquired dimensions which render them accessible to our means of investigation. The time of their appearance, following the same order according to which they succeed each other, presents numerous differences by which we explain those so frequently reproduced in the eruption of the teeth. They are such that, to confine myself to the results furnished by anatomical observation, I have only been able to assign general terms

* Dictionnaire de Médicine, ou Répertoire Gènéral des Sciences Médicales, t. x. article *Dentition*, p. 99, 1835.

to them, more or less approximative. There are unquestionably those difficulties, joined to those which arise from the delicacy of the parts which have to be studied, which, reasonably enough to us, occasion the discords among anatomists on this subject. I should not blame them for this if I were able to fall in with a small number of authors who have verified for themselves the facts which they describe—the greater part seeking to disguise their plagiarism by greater or less modifications. For myself, I prefer the author of the *True Anatomy of the Teeth*; he copies a celebrated anatomist at least for the accuracy, and leaves to future writers to ask him if anatomy is not indebted to Urbain Hèmard for the beautiful researches upon the dental follicles which had been before made by Eustachius.*

Everard Home, and particularly Blake, has emitted some ideas on the formation of the follicles of the large molars which we cannot permit ourselves to pass in silence. According to their dicta, the follicle of each of these teeth may be regarded in turn as the result of an elongation of the sac which precedes it. Thus, in principle, the membranes of the posterior milk molar and those of the first permanent large molars are intimately united together and continue in the same alveolus; but in proportion as this last is developed, and as the jaws extend, they are placed in a particular alveolus which communicates with the preceding by an opening traversing their common partition. Their capsule then throws out a prolongation for the second large molar; this is formed at first in the alveolus which is common with the first, then they are separated by a bony partition to constitute in its turn, and in the same manner, the sac of the third large molar or wisdom tooth.

This explanation, ingenious as it has seemed to some physiologists, and well as it appears under some circum-

*Bartholomæi Eustachii Tractatus de Dentibus, 1726.

stances, is nothing more than a pure hypothesis ; one must have studied nature with little care to attribute this kind of caprices to her by which she would deviate from the general laws which she has imposed upon herself. While the question as to the origin of the permanent large molars was agitated by Home and Blake, I was seriously occupied in my first works about the germs of all of the teeth and I had then arrived at a safe induction.

Two essential conditions are attached to the whole dental organism, to wit:—to be, punctually, the seat of a work of production ; and to be always in connection with the mucous system. But what are these connections? All authors who have written upon the germs of the teeth, have described them as created and growing in the cavity of the maxillary bones, very near to the gums with some parts of which they are in immediate contact, and with others by the prolongation of their capsules. Well, this opinion, which I entertained for a certain time, I cannot now admit ; I nevertheless believe that a most intimate connection exists between the teeth and the tissue of the gum.

I mentioned above that, in following the nervous filament intended for the wisdom tooth in the jaws of young children, I had found a species of swelling formed by the gum at the place where it terminates. Influenced by received ideas and encountering in the body the configuration more peculiar to the ganglions than the characteristics which I had been accustomed to find in the follicles nearly at the period of their first appearance, I hesitated, and, in the doubt, I dared not affirm that I had really found the follicles of the wisdom teeth. However these facts were of too much importance to allow me to rest tranquilly in the doubt ; I could neither address myself to the germs of the teeth of first dentition nor to those of the permanent incisors or canines ; the parts which should be submitted to these investigations appeared at too early a period of fœtal life, and, moreover, they are so small and so delicate, that I was compelled to run the risk of seeking that which did not exist, or of failing to perceive that which did exist. For these reasons I directed my researches to the second large permanent molars, the follicles of which, as I have said, appear at a period when the jaws are sufficiently large. If we macerate the inferior maxillary bone of a fœtus, or that of a young child just after birth, in an acidulated solution, we may discover the inferior dental nerve about half a millimetre in diameter at the beginning of its canal. This nervous filament takes a retrograde direction; at first from below upward and from the front backward, and afterwards from behind frontward, making the circuit of the internal border of the posterior face of the follicle of the first large permanent molar so as to describe a loop, the concavity of which is turned frontward, the convexity corresponding behind to the ramus of the jaw; from this point the filament joins the gum and very distinctly presents a spheroidal swelling situated at the bottom of, and within, the follicle of the first large permanent molar. If we examine the same parts at a more advanced period of dentition—in the course of the third year—we may remark that the dental cord, which we have described, is larger and separated from the follicle of first permanent large molar by an ossified partition. Its volume sensibly increases as it approaches the gum from which it is separated by a little membraneous cord six millimetres in length, of a lively red color and possessing a marked pulpy consistence. The appearance of this little body announces the work which is going on in the germ contained in the gum and from whence the follicle of the second permanent large molar emerges.

From these facts* I feel justified in concluding that the germs of the teeth primitively exist in a rudimentary state in the gums. They remain stationary until the vital activity, with varied power, animates them to an exertion of their proper functions.

* Many of these dissections have been made under the eye of one of my respected colleagues, Dr. Hugnier, whose name is familiar to the readers of anatomical works.

Thus, after having proved in 1823, the analogy which exists between the teeth and corns, in respect to the organic acts which concur in their formation and growth, I came to show that the same analogy may be discovered in the primitive situation of the parts charged with the accomplishment of these acts, and that the germs of the teeth, for the mucous system, like bulbs for the cutaneous system, both are an integral part of these organic systems. The demonstration is now complete. But what has guided me? Physiological induction, I should have said were I not afraid of making use of an expression which might seem pretentious.

Surrounded by difficulties, in my first works, this voice, conducted me to the truth. I saw that some teeth were only partially covered by enamel whilst others were wholly covered; the development of some was invariably limited; others possessed the faculty of continued reproduction, also other productions of the tegumentary system; I found teeth simple and teeth complex, etc. I was struck by these dissimilarities and contrasts; I observed them but I did not wish, like my predecessors, that they should operate as lines of demarcation separating the teeth from the other phenomena of nature. Far from it; I accepted them as diverse manifestations of the same work. I studied this work and the organs charged with the execution of it and the laws which directed them. From thence all of these exterior forms, all of these combinations, diversification and subsequent complications under which they were hidden, enrolled themselves, simply and naturally, and found their explication as their necessity, in the first arrangement of these organs and in the exercise of the same physiological laws to which they are equally and invariably submitted.

I arrived, without help, at the practice of those principles of a celebrated school of philosophic or comparative anatomy. This school, which the genius of Aristotle had preshadowed, and the route of which was traced out by Vicyd'Azyr, was then listened to with defiance when not with disdain. It was guilty of that fault, so difficult to forgive, of attacking universally received ideas, and, still more unhappily, of encountering as adversaries, the most illustrious and powerful men of science. During the prelude to the struggle which latter it had to sustain, I arose, on my part, against the march followed by comparative anatomy in the general study of the teeth; I showed that it attached itself too exclusively to exterior, secondary characters, and that it did not occupy itself enough with the organ which is the cause of them, and with the anatomical modifications to which it submits; that above all, it had the grave fault of isolating facts and of tracing the separate pictures instead of reuniting them by organic bonds into one and the same system ; and in a word, that considered from this point of view it was rather a method of classification than a science, properly speaking. I did more, I laid down the limits which should guide naturalists in the order of distribution of the teeth, and I called their attention to a work on a new subject. Since then several works have appeared on this subject and their authors have not found it necessary to renounce their ancient convictions; I respect them, but I must defend my own.

I now revert to some of the propositions formularized in my preceding memoirs. While they have not been positively assailed, I have nevertheless thought it necessary to develop them to some extent, that they may be the more readily comprehended, and the more scientifically valuable. Besides, another motive engaged me ; I, unhappily, found myself in opposition to the opinions contained in a work protected by one of the most celebrated names of our age. In passing these opinions in silence, I should fear to show myself indifferent before the authority which they borrow from the elevated position of their author, or seem to sacrifice the interest of science to personal considerations and repugnance. Thus, in discussing them, am I less happy in being able to render my homage and at the same time in accomplishing a duty?

The questions which I shall examine, relate to the definition and to the divisions of the teeth, and to the characteristics of the roots.

### *A. Definition of the Teeth.*

It would perhaps appear more convenient in a science of observation—in order to proceed methodically—to remit its definitions to the concluding part of the work, as they would thus appear as the necessary consequences of the facts reported—a succinct résumé of the science. By this method, I conceive, they would become less free, for they would be obliged to accord with the facts; but these in their turn, would command a more serious examination, and acquire, in consequence, greater authority.

Thus the teeth having been regarded as bone, they have naturally been defined in accordance with this opinion. The characteristics of bone were supposed to be known, and these were applied to the teeth. They were only distinguished by certain peculiarities accessory to their situation and uses. In this manner, the name of *tooth* conveyed a certain precise, determinate notion, which was far from correct. Some writers, it is true, at different times, emitted doubts as to the osseous nature of the teeth; but these doubts were allowed to remain sterile. Hunter* has the merit of having first established the correct view. His genius advanced beyond the limits which science had reached in assigning their true characteristics to the teeth. Nevertheless, as I have elsewhere remarked, Hunter's theory was incomplete and did not lead to a positive determination of the question. He directed his attention at too late a period to comparative anatomy and experimental physiology, to completely demonstrate what the teeth are; productions of the mucous system resembling all of those which rise from the

* *Œuvres Completes*, trans. by Richelot, Paris, 1841, t. 11, 8vo.

skin. This consequence of facts, to the attainment of which I have devoted twenty-five years, I arrived at, as the basis of the definition of the teeth, in a memoir read before the Medical Society. I then stated, as a fundamental principle, that an organ should always be defined by its most constant and essential characteristics, and which should be distinctive. In this respect, it would appear that precise definitions are the best introduction to descriptive works, as they facilitate the intelligence in apprehending the subject. The mind is thus enabled to acquire a spirit of generalization in the consideration of gradually unfolding details without embarrassment.

Thirty years after, the teeth were defined in one of the most important works on comparative anatomy,* as follows: "mechanical instruments, harder than bone, placed at the entrance of the alimentary canal in vertebrates, for the purpose of seizing, cutting, tearing, crushing or bruising the *nutritive* substances, previous to their transmission from the mouth to the esophagus, or in order to permit of easy deglutition. They also serve some animals as an offensive or defensive weapon."

We certainly cannot accuse this definition of being laconic; but since this has been avoided, have we not a right to ask for some more precise description of what the teeth are, and what place they occupy among the divers systems of our economy. Is it true that, after all the works which have been published on these organs during thirty years, comparative anatomy is content to consider them merely as mechanical instruments harder than bone? Shall we deny the name of teeth to those dental productions which are less hard than bone in certain animals? Do these delinquencies satisfy any anatomical character? By no means. Any physiological character? Still less.

All definitions of the teeth are encumbered by two principal vices; they repose less upon the real nature of these

* G. Cuvier *Lecons d'Anatomie Comparée*, 2d ed. 1835, note by Duverny, t. iv, 1st part, p. 197.

productions, studied in themselves, than upon the designation of certain respects, true or apparent, of analogy or of difference, with a tissue upon which science has not perhaps yet pronounced definitely; but they are also defective in abstracting the most important part of the tooth; its productive organ; of that which precedes it, and has to do with its future constitution; they exclusively draw from physical or chemical qualities, that is to say, of secondary characteristics, which are of less value in an anatomical determination.

In this these definitions only translate the spirit of the science which has made them. It is necessary to repeat, for comparative anatomy, that the teeth have nearly always been an instrument of classification rather than a subject of physiological investigation; it is this which has caused science to remain uncertain so long in the presence of phenomena which were able to throw light upon it. I know perfectly well that they are not limited to certain exterior signs; that to render these definitions applicable to the teeth, they comprehend the uses which these organs have in different animals; but is this last indication more satisfactory than the first? I think not. How is it that they have failed to give a true signification to the organism, after the part it plays in the functions of our economy? Has not Geoffroy Saint Hilaire always and for a long time insisted upon comprehending in a definition of an organ the functional acts which it is able to accomplish?

The teeth serve most generally for mastication; but are they not of service in any other respect? Do they not vary according to the needs, tastes, and habitudes, and in a certain elevated region in the scale of animals, according to intelligence? For some they are proper instruments for seizing or retaining their prey; for others as defensive or offensive weapons; and to others again, they assist in the articulations of the voice, etc. When we speak of their uses, should we omit the most physiological of all, namely, the tactile faculty they possess, by which they are able to apprehend the physical qualities of bodies submitted to their action? Should we not know that these are the teeth and the rank they occupy in the organism?

The fundamental character of the teeth, that which reposes upon their essential nature, upon the mode following which they form and grow, upon the intimate properties of the parts which constitute them, the character, finally, without which we are unable to conceive of their existence, is, that they are a production of the mucous system. Whence it follows that the composition of these substances, their situation either at the entrance of the digestive tract or more deeply seated, and their use for mastication or any other purpose; all these are of little importance, as accessory traits, as they are but the complement of the principal definition.

It does not become me to judge this definition, but I do not fear to say that it expresses at least a clear and precise idea. The name of *teeth* receives a positive sense, which places them among the productions of the tegumentary system and forever separates them from the osseous tissue. And in this announcement it would be improper to suppose it a purely speculative opinion. This would be gravely misapprehending me. In sciences of observation the truth is fortified by new truths, as error increases by the propagation of error. If we recognize in the teeth certain resemblances to bone, it would be necessary to conglomerate into one body of doctrine all the facts which are attached to the history of these organs, and one would be authorized to generalize and develop from each of them the deductions to which they would conduct. Since then, observation and theory would become common to all of them; science could not advance some without equally promoting others. But if, on the contrary, we admit that the teeth should be assimilated to the other productions of the tegumentary system, all these questions would be found to be transposed to another basis; we should be obliged to renounce the deceptive analogies by clearing them up with new lights which new considerations would suggest ; we would search for them in the circumstances where they are manifested, and in the situation in which they would be placed, so that an important discovery could not be made upon one point without benefiting others. Thus, and the researches placed at the head of this work prove it—I have often been able to remove certain difficulites which have occurred to me by this comparative study.

(To be continued.)

